# Supplementary material for: Orthogonal replication with optogenetic selection evolves yeast JEN1 into a mevalonate transporter
Source: Mol Syst Biol. 2025 Jun 11;21(9):1190–213. doi: 10.1038/s44320-025-00113-5 (PMC12405511; doi:10.1038/s44320-025-00113-5)
Supplement: Supplementary file 1 — Appendix [file 44320_2025_113_MOESM1_ESM.pdf]

Appendix for

# Orthogonal replication with optogenetic selection evolves yeast *JEN1* into a mevalonate transporter.

Scott A. Wegner[1], Virginia Jiang[2], Jeremy D. Cortez[1], & José L. Avalos[1-5 *]

1. Department of Molecular Biology, Princeton University, Princeton, NJ 08544, USA.
2. Department of Chemical and Biological Engineering, Princeton University, Princeton, NJ 08544, USA.
3. The Omenn-Darling Bioengineering Institute , Princeton University, Princeton, NJ 08544, USA
4. The Andlinger Center for Energy and the Environment, Princeton University, Princeton, NJ 08544, USA.
5. High Meadows Environmental Institute, Princeton University, Princeton, NJ 08544, USA.

Corresponding author address – 101 Hoyt Laboratory, 25 William Street Princeton, NJ 08544.

[*]Correspondence: javalos@princeton.edu

**Table of contents:**

**Appendix Figure S1.** Compatibility of OrthoRep with the OptoMEV optogenetic strain ………. 3

**Appendix Figure S2.** OptoREP mutagenesis of eight independent OptoRep-*JEN1t* lineages under semi-permissive light conditions ……………………………………………………………….. 4

**Appendix Figure S3.** PyRx docking and Rosetta modeling of mevalonate binding to Jen1p …….. 5

**Appendix Figure S4.** Characterization of strains expressing JEN1t mutants for their ability to convert exogenous mevalonate into farnesene ………………………………………………………. 6

**Appendix Figure S5.** Mevalonate feeding in high farnesene production strains lacking (SAWy726) or expressing (SAWy727) JEN1tY180G ……………………………………………………….... 7

**Appendix Figure S6.** Stability and copy number of Jen1ptY180G ……….……………….……. 8

**Appendix Sequence S1.** ΔNT94ΔCT33 Truncated *JEN1* (*JEN1t*) …..…………………..……..... 9

**Appendix Table S1.** Growth rate of optoMEV and parent strains ….…………………..……..... 9

**Appendix Table S2.** OrthoREP compatibility with optogenetics ……………………..………. 9

**Appendix Table S3.** Percent growth rate of 2%100 µmol m-2 sec-1 pulse conditions ..……..…... 9

**Appendix Table S4.** Growth rates in non-permissive light conditions, in media containing 10 mM mevalonate, of OptoMEV expressing JEN1t or JEN1tY180C from a CEN/ARS plasmid ………..…10

**Appendix Table S5.** Plasmids used in this Study ……………..……..…………………………. 11

**Appendix Table S6.** Yeast strains used in this Study …………………….……..……..………...12

**Appendix Table S7.** Relevant primers in this study…………………..…………………….…. 15

**Appendix References**……………………………………………………………………….. 16

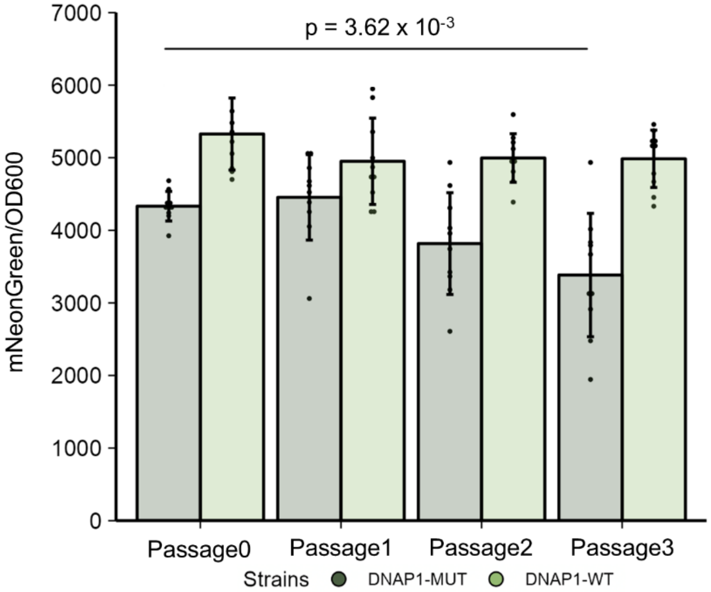


**Appendix Figure S1.** Compatibility of OrthoRep with the OptoMEV optogenetic strain. To verify that OrthoRep is functional in OptoMEV, we introduced the OrthoRep components (p1, p2, and TP-DNAP1), with mNeonGreen in p1 into the optoMEV strain (resulting in strain OrthoRep-mNG). Passaging of OrthoRep-mNG was done using either wild-type (SAWy696; DNAP1-WT) or error-prone TP-DNAP1 polymerase (SAWy678; DNAP1-MUT). We found that the fluorescence of mNeonGreen encoded in the p1 of OrthoRep-mNG deteriorates within three passages when expressing the mutant TP-DNAP1 but not the wildtype (Appendix TableS2), suggesting that mutagenesis of this reporter by OrthoRep is effective in the optogenetic OptoMEV strain. The initial fluorescence of an overnight culture was measured (Passage 0), along with average bulk fluorescence for three 24-hour passages (Passage 1, Passage 2, and Passage 3). Bars represent the average and error bars the standard deviation of 10 independent lineages. Data analysis was performed with a two-sided Mann-Whitney U test.

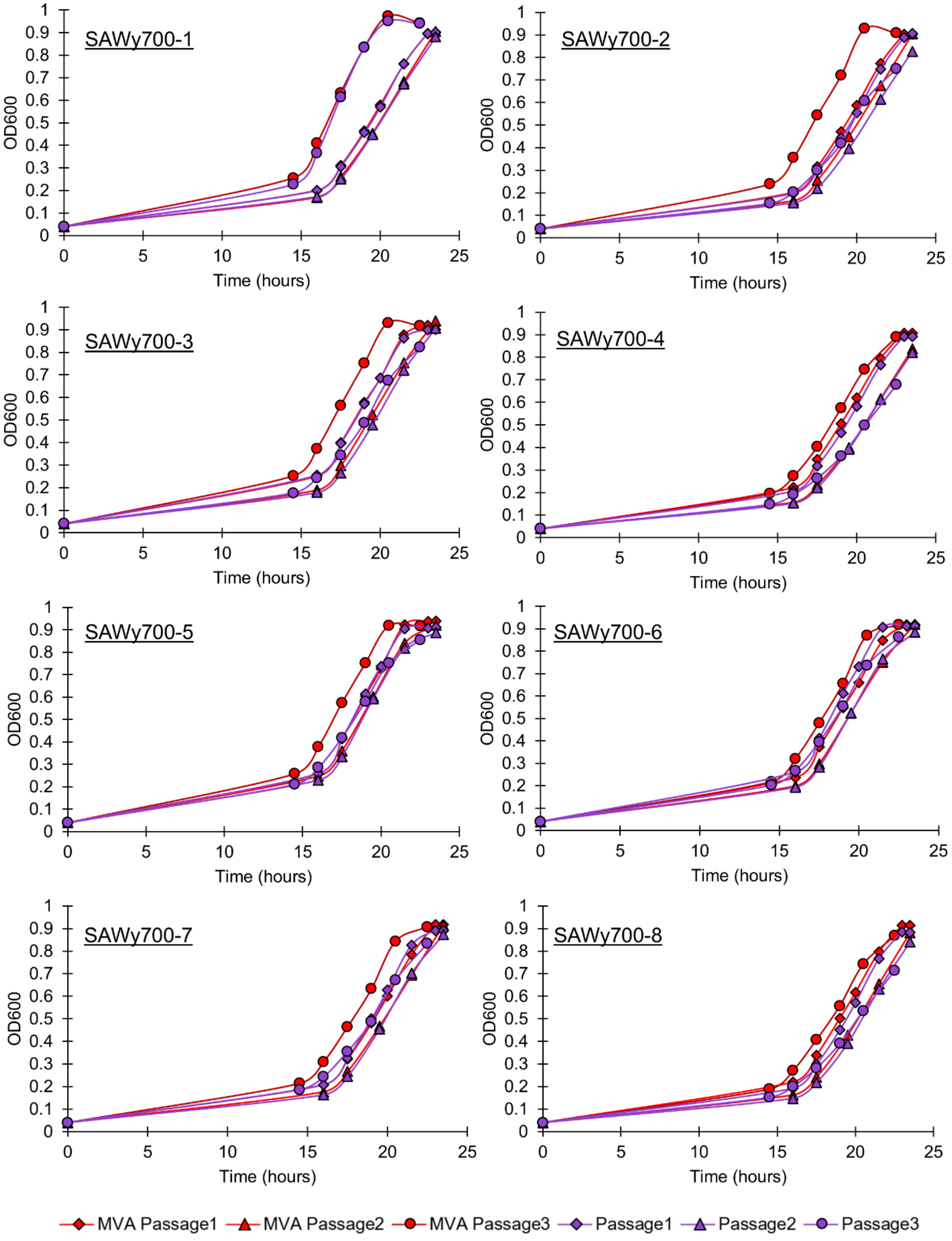


**Appendix Figure S2.** OptoREP mutagenesis of eight independent OptoRep-*JEN1t* lineages under semi-permissive light conditions. Growth profiles for each independent lineage subjected to OptoREP mutagenesis, with each passage performed under 2% 100 µmol $m^{-2}$ $sec^{-1}$ light conditions in 100 s forcing periods. MVA refers to growth conditions supplemented with 10 mM mevalonate. Optical density measurements are presented in Tecan units (see Methods).

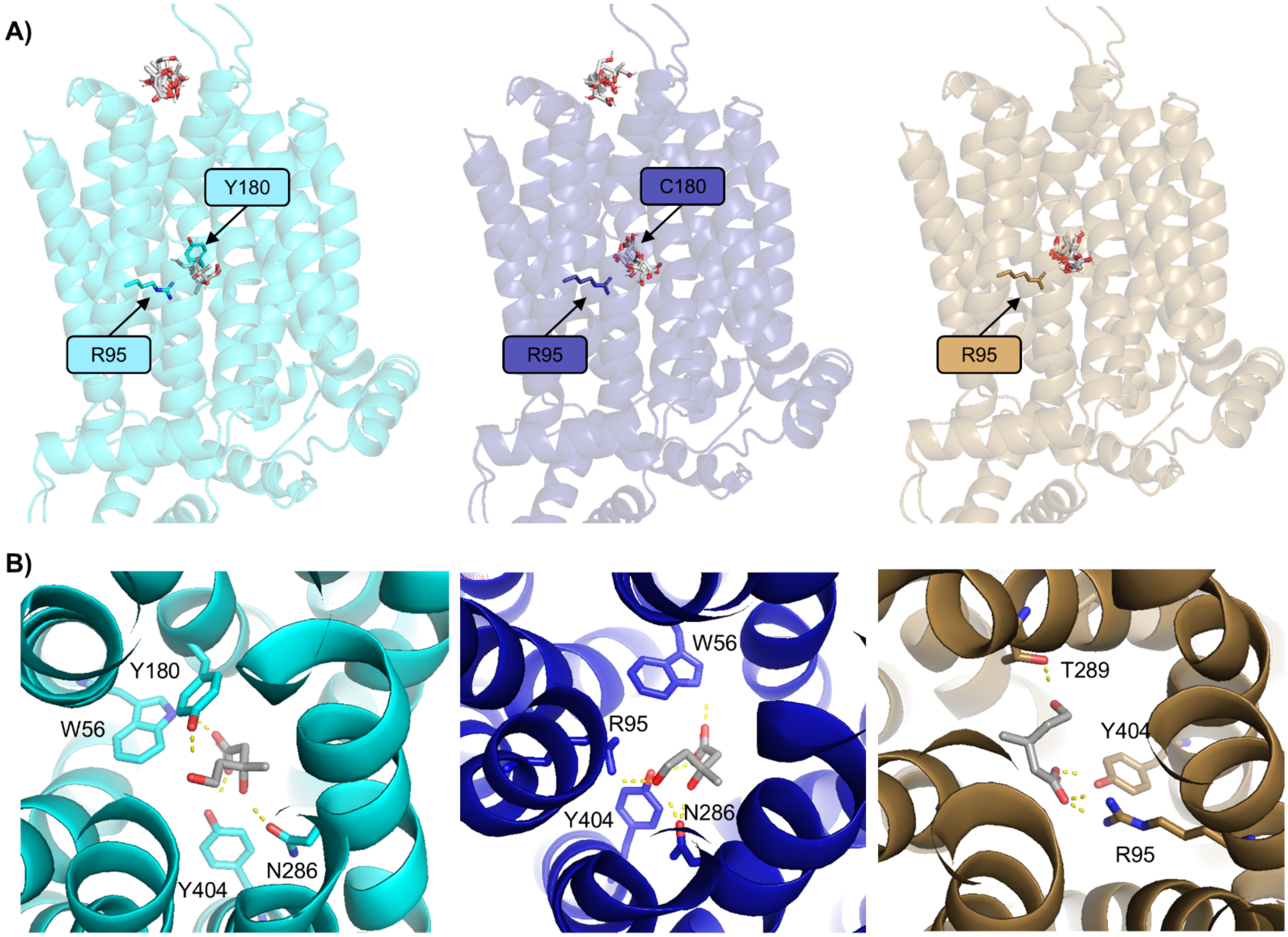


**Appendix Figure S3.** PyRx docking and Rosetta modeling of mevalonate binding to Jen1p. **A)** The wild-type Jen1p transporter (left), Jen1p$^{Y180C}$ (middle), and Jen1p$^{Y180G}$ (right) are shown with the critical R95 residue (equivalent to R188 in the full length Jen1p) and the mutated Y180 residue (equivalent to Y273 in the full length Jen1p). Nine different simulated mevalonate conformations are shown for each Jen1p variant. Computational structure models of mevalonate bound to wild-type JEN1t, JEN1t$^{Y180C}$, and JEN1t$^{Y180G}$ are available on Model Archive (modelarchive.org) with accession codes ma-39yos, ma-hnfca, and ma-dqvhp, respectively. **B)** Mevalonate conformations with the lowest interfacial energy of binding to the putative binding pockets of each Jen1p variant shown in **(A)**. These correspond to the most favorable binding conformations, with predicted hydrogen bonds and salt bridges shown as dashed yellow lines.

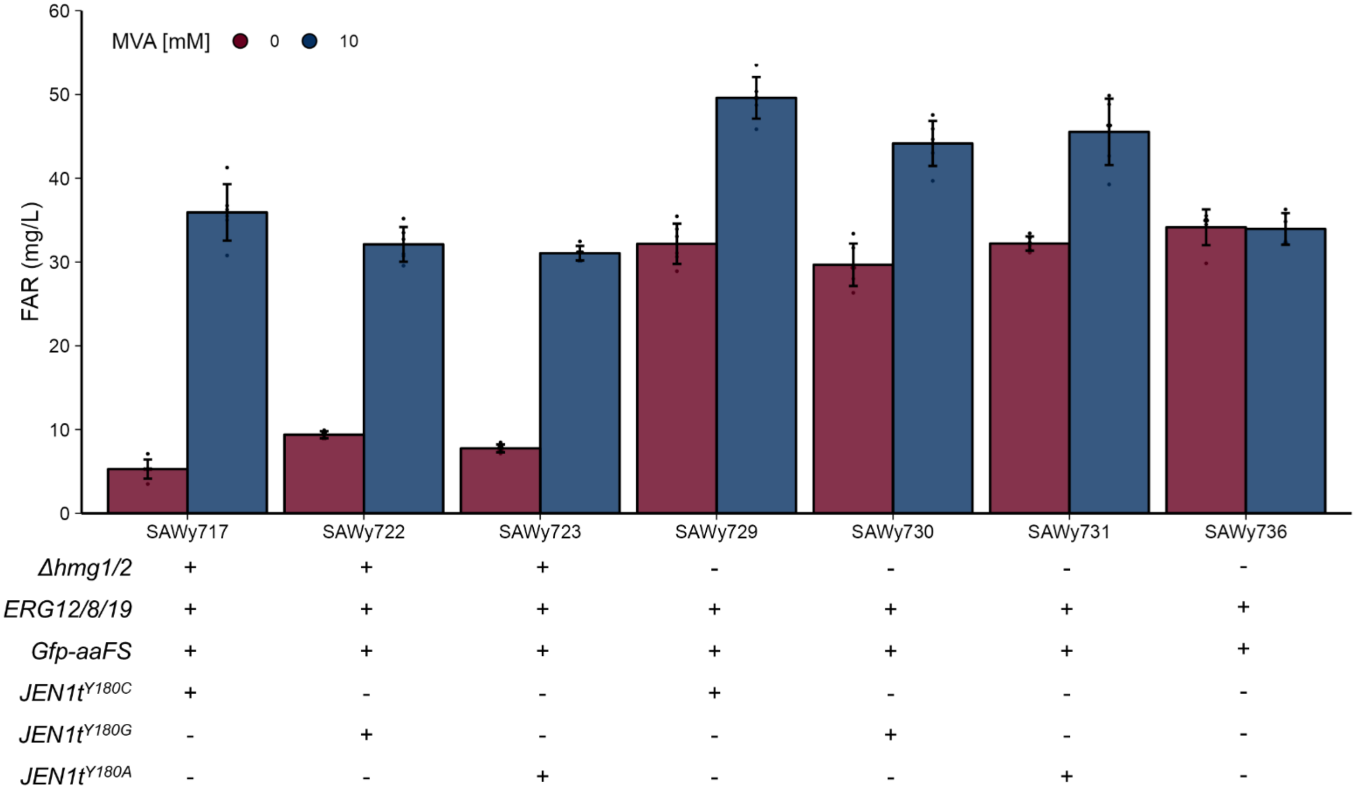


**Appendix Figure S4.** Characterization of strains expressing *JEN1t* mutants for their ability to convert exogenous mevalonate into farnesene. HMG-null strains fully deficient in mevalonate production (SAWy717, SAWy722, SAWy723) were compared alongside strains with basal levels of mevalonate pathway flux (SAWy729- SAWy731, SAWy736). All strains contain a single-copy overexpression of the lower MVA pathway (*ERG12*, *ERG8*, *ERG19*) with a GFP-stabilized farnesene synthase (aaFS). High-density fermentations were performed in SC-URA, pH5, 2% glucose media with or without 10 mM mevalonate. Overnight cultures of HMG-null strains were grown with 2 mM mevalonate to allow growth. SAWy736 contains an empty CEN/ARS vector. Average and individual data points for 6 biologically independent replicates are shown with error bars showing the standard deviation for each measured timepoint.

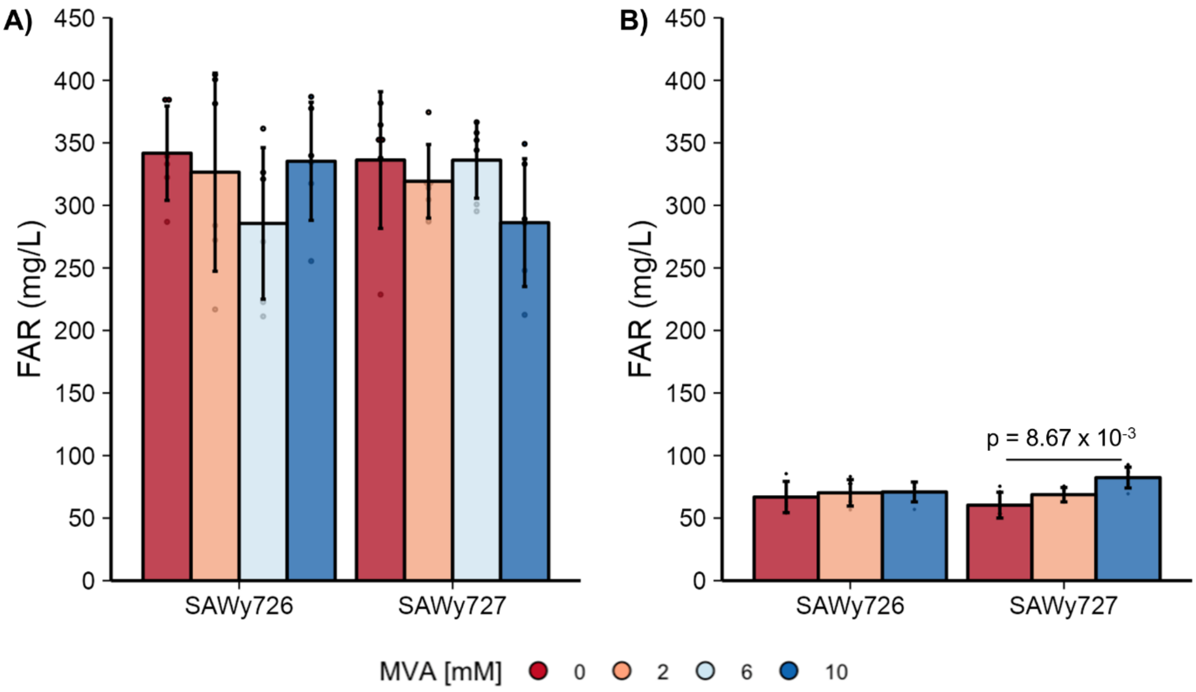


**Appendix Figure S5.** Mevalonate feeding in high farnesene production strains lacking (SAWy726) or expressing (SAWy727) *JEN1t*$^{Y180G}$. **A, B)** Farnesene production by feeding different concentrations of mevalonate (MVA) to these relatively high farnesene-producing strains, either with **(A)** or without **(B)** methionine-induced *ERG9* repression. Average and individual data points for 6 biologically independent replicates are shown with error bars showing standard deviation. Data analysis was performed with ANOVA with Tukey correction for all panels. $^{*}$ $p< 0.05$, $^{**}$ $p < 0.01$, $^{***}$ $p < 0.001$, $^{****}$ $p < 0.0001$.

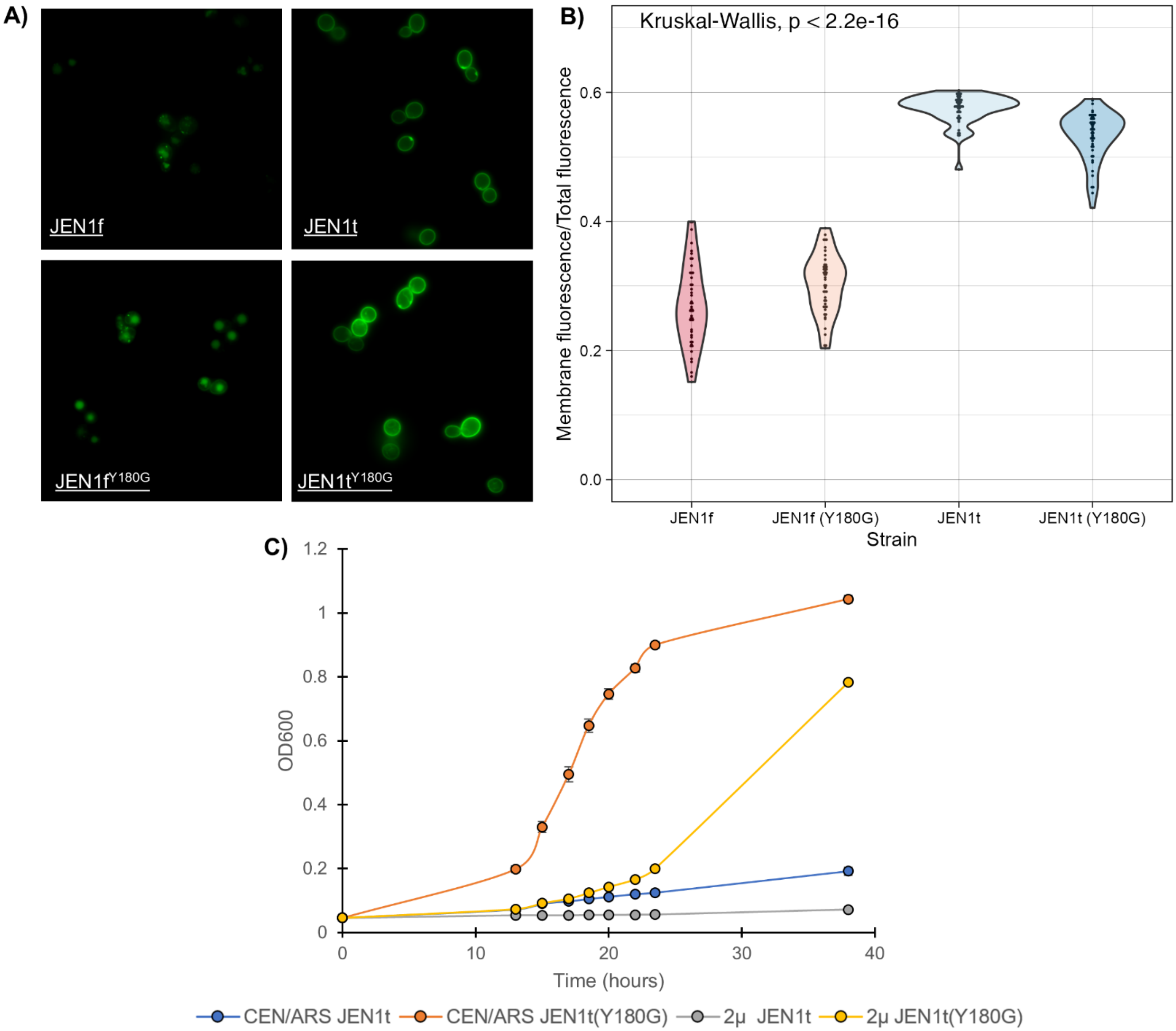


**Appendix Figure S6.** Stability and copy number of Jen1p*t*$^{Y180G}$. **A)** Visualization of GFP-labeled full length Jen1p (*JEN1*f) versus truncated (*JEN1*t) constructs bearing either the Y180 wildtype or mutant Y180G sequence. Strain yTK29 was transformed with the different *JEN1* constructs, and each derived strain was grown overnight in glucose-containing media, which promotes internalization of the full length Jan1p constructs. **B)** Quantification of GFP fluorescent signal localized to the plasma membrane for each Jen1p construct. This analysis yielded results consistent with previously reported values for the stability of the truncated Jen1pt constructs at the plasma membrane (n = 48 cells/condition). Interestingly, the Y180G mutation slightly reduces membrane fluorescence (0.532 ± 0.0385); however, this reflects a less than 10% difference to the wild-type sequence. **C)** Growth of the optoMEV strain expressing either wildtype *JEN1t* or *JEN1t*$^{Y180G}$ using either low-copy (CEN/ARS) or high-copy (2μ) expression vectors. Growth assays were performed in non-permissive light conditions with media supplemented with 10 mM mevalonate. Optical density measurements are presented in Tecan units (see Methods).

**Appendix Sequence S1. ΔNT94ΔCT33 Truncated *JEN1* (*JEN1t*)(Barata-Antunes et al., 2022).**

MKPNLSAASIKSYALTRFTSLLHIHEFSWENVNPIPELRKMTWQNWNYFFMGYFAWLSAAWAF
FCVSVSVAPLAELYDRPTKDITWGLGLVLFVRSAGAVIFGLWTDKSSRKWPYITCLFLFVIAQLC
TPWCDTYEKFLGVRWITGIAMGGIYGCASATAIEDAPVKARSFLSGLFFSAYAMGFIFAIIFYRAF
GYFRDDGWKILFWFSIFLPILLIFWRLLWPETKYFTKVLKARKLILSDAVKANGGEPLPKANFKQ
KMVSMKRTVQKYWLLFAYLVVLLVGPNYLTHASQDLLPTMLRAQLGLSKDAVTVIVVVTNIG
AICGGMIFGQFMEVTGRRLGLLIACTMGGCFTYPAFMLRSEKAILGAGFMLYFCVFGVWGILPI
HLAELAPADARALVAGLSYQLGNLASAAASTIETQLADRYPLERDASGAVIKEDYAKVMAILTG
SVFIFTFACVFVGHEKFHRDLSSPVMKKYINQVEEYEADGL*

**Appendix Table S1.** Growth rate of optoMEV and parent strains.

| Strain | Description | Light ($hr^{-1}$) | Light + MVA ($hr^{-1}$) | Dark ($hr^{-1}$) | Dark + MVA ($hr^{-1}$) |
|---|---|---|---|---|---|
| SAWy119 | WT | 0.286 ± 0.033 | 0.255 ± 0.032 | 0.271 ± 0.007 | 0.281 ± 0.007 |
| SAWy518 | *Δhmg2* | 0.253 ± 0.024 | 0.232 ± 0.025 | 0.268 ± 0.006 | 0.271 ± 0.012 |
| SAWy524 | optoMEV | 0.251 ± 0.020 | 0.245 ± 0.017 | 0.021 ± 0.010 | 0.047 ± 0.006 |
| SAWy644 | optoMEV, *Δjen1* | 0.263 ± 0.008 | 0.219 ± 0.009 | 0.032 ± 0.007 | 0.058 ± 0.013 |

**Appendix Table S2.** OrthoREP compatibility with optogenetics.

| Strain | Passage | Mean Fluorescence (a.u.) | Fluorescence (% Passage 0) |
|---|---|---|---|
| SAWy678 (DNAP1-MUT) | 0 | 4332.093 ± 193.582 | 100 ± 4.7 |
| SAWy678 | 1 | 4454.602 ± 558.962 | 102 ± 13.6 |
| SAWy678 | 2 | 3817.147 ± 665.367 | 88 ± 16.2 |
| SAWy678 | 3 | 3383.968 ± 806.252 | 78 ± 19.6 |
| SAWy696 (DNAP1-WT) | 0 | 5326.969 ± 470.798 | 100 ± 9.3 |
| SAWy696 | 1 | 4950.947 ± 564.072 | 93 ± 11.1 |
| SAWy696 | 2 | 4996.516 ± 317.382 | 93 ± 6.3 |
| SAWy696 | 3 | 4985.512 ± 375.321 | 94 ± 7.4 |

**Appendix Table S3.** Percent growth rate of 2%100 μmol $m^{-2}$ $sec^{-1}$ pulse conditions.

| | Overnight Light | 100% Light | 2% Light |
|---|---|---|---|
| 0.01 Inoculation | Pulsed | 100 ± 2.48 | 73.7 ± 10.6 |
| | Full | 100 ± 2.21 | 79.8 ± 7.0 |
| 0.1 Inoculation | Pulsed | 100 ± 2.03 | 87.1 ± 2.0 |
| | Full | 100 ± 4.80 | 92.2 ± 4.9 |

**Appendix Table S4.** Growth rates in non-permissive light conditions, in media containing 10 mM mevalonate, of OptoMEV expressing *JEN1t* or $JEN1t^{Y180C}$ from a CEN/ARS plasmid.

| Strain | Dark + MVA |
|---|---|
| SAWy524 + empty plasmid | 0.013 ± 0.003 $hr^{-1}$ |
| SAWy524 + JEN1t (WT) | 0.023 ± 0.002 $hr^{-1}$ |
| SAWy524 + JEN1t (Y180C) | 0.203 ± 0.003 $hr^{-1}$ |

**Appendix Table S5.** Plasmids used in this Study.

| **Plasmid** | **Contents** | **Source** |
|---|---|---|
| Ec318 | CEN/ARS *HIS4*, $P_{REV1}$_WT-TPDNAP1_$T_{ADH1}$ | (Ravikumar et al., 2018) |
| Ec633 | CEN/ARS *HIS4*, $P_{REV1}$_MUT-TPDNAP1-4-2_$T_{ADH1}$ | (Ravikumar et al., 2018) |
| EZL571 | *HIS3*, $P_{TEF1}$_VP16-EL222_$T_{CYC1}$, $P_{C120}$_GAL80_$T_{ADH1}$, $P_{ADH1}$_GAL4_$T_{ACT1}$, $P_{C120}$_GAL80_$T_{ADH1}$ | (Zhao et al., 2020) |
| JMCp10 | bleMX6, $P_{GPD1}$_mvaE_$T_{ADH1}$, $P_{TEF}$_mvaS_$T_{ACT1}$, $P_{PGK}$_Acs(L641p)_$T_{CYC1}$ | (Wegner et al., 2021) |
| pV1382_ERG9 | CEN/ARS *URA3*, $P_{TEF1}$_caCas9_$T_{CYC1}$, sgRNA_ERG9 | (Wegner et al., 2021) |
| pV1382_HMG1 | CEN/ARS *URA3*, $P_{TEF1}$_caCas9_$T_{CYC1}$, sgRNA_HMG1 | |
| pYZ125 | CEN/ARS *URA3* empty vector | (Zhao et al., 2018) |
| SAWlig100 | *URA3*, $P_{GPD1}$_mvaE_$T_{ADH1}$, $P_{TEF1}$_mvaS_$T_{ACT1}$, $P_{PGK1}$_Acs(L641p)_$T_{CYC1}$ | |
| SAWlig120 | *LEU2*, $P_{GPD1}$_ERG19_$T_{CYC1}$, $P_{TEF1}$_ERG8_$T_{ACT1}$, $P_{PGK1}$_ERG12_$T_{ADH1}$ | |
| SAWlig204 | *TRP1*, $P_{GPD1}$_ERG20_$T_{CYC1}$, $P_{TEF1}$_IDI1_$T_{ACT1}$, $P_{GPD1}$_aaFS_$T_{ADH1}$ | |
| SAWlig232 | *LEU2*, $P_{GPD1}$_aaFS_$T_{ADH1}$ | |
| SAWlig260 | bleMX6::$P_{GPD1}$_aaFS_$T_{ADH1}$ | |
| SAWlig424_MUT | *LEU2*, $P_{REV1}$_TPDNAP1-4-2_$T_{ADH1}$ | |
| SAWlig424_WT | *LEU2*, $P_{REV1}$_TPDNAP1_$T_{ADH1}$ | |
| SAWlig427 | p1 *URA3*, $P_{10B2}$_mNeonGreen_A75_Ribozyme_$T_{ADH1}$ | |
| SAWlig500 | p1 *URA3*, $P_{10B2}$_JEN1t_A75_Ribozyme_$T_{ADH1}$ | |
| SAWlig523 | CEN/ARS *URA3*, $P_{GPD1}$_JEN1t(WT)_$T_{ADH1}$ | |
| SAWlig548 | CEN/ARS *URA3*, $P_{GPD1}$_JEN1t(Y180C)_$T_{ADH1}$ | |
| SAWlig568 | *LEU2*, $P_{TEF1}$_GFP_aaFS_$T_{SSA1}$, $P_{GPD1}$_ERG19_$T_{CYC1}$, $P_{TEF1}$_ERG8_$T_{ACT1}$, $P_{PGK1}$_ERG12_$T_{ADH1}$ | |
| SAWlig592 | 2μ *URA3*, $P_{GPD1}$_JEN1t wt_$T_{ADH1}$ | |
| SAWlig593 | 2μ *URA3*, $P_{GPD1}$_JEN1t Y180G_$T_{ADH1}$ | |
| SAWlig594 | CEN/ARS *URA3*, $P_{GPD1}$_JEN1f_GFP_$T_{CYC1}$ | |
| SAWlig594-mut | CEN/ARS *URA3*, $P_{GPD1}$_JEN1f (Y180G)_GFP_$T_{CYC1}$ | |
| SAWlig595 | CEN/ARS *URA3*, $P_{GPD1}$_JEN1t_GFP_$T_{CYC1}$ | |
| SAWlig595-mut | CEN/ARS *URA3*, $P_{GPD1}$_JEN1t (Y180G)_GFP_$T_{CYC1}$ | |
| SAWlig598 | CEN/ARS *URA3*, $P_{GPD1}$_JEN1t(Y180G)_$T_{ADH1}$ | |
| SAWlig599 | CEN/ARS *URA3*, $P_{GPD1}$_JEN1t(Y180A)_$T_{ADH1}$ | |
| SAWlig600 | CEN/ARS *URA3*, $P_{GPD1}$_JEN1t(Y180T)_$T_{ADH1}$ | |
| SAWlig602 | *LEU2*, $P_{TEF1}$_GFP_aaFS_$T_{SSA1}$, $P_{GPD1}$_ERG19_$T_{CYC1}$, $P_{TEF1}$_ERG8_$T_{ACT1}$, $P_{PGK1}$_ERG12_$T_{ADH1}$, $P_{GPD1}$_JEN1t (Y180G)_$T_{ADH1}$ | |

**Appendix Table S6.** Yeast strains used in this Study.

| Strain Name | Genotype | Source |
|---|---|---|
| CEN.PK2-1C | MATa *his3Δ1 leu2-3_112 trp1-289 ura3-53* | (Entian & Kötter, 2007) |
| yAR-406 | MATa *can1 his3 leu3Δ0 ura3Δ0 HIS4 flo1Δ*::KanMX p0 + p1 + p2 | (Ravikumar et al., 2018) |
| yTK29 | CENPK2-1C *trp1*::*TRP1*, $P_{TPI1}$_SS-mCherry-HDEL_$T_{CYC1}$ | (Kichuk et al., 2024) |
| yEZ44 | CEN.PK2-1C *gal80Δ*::lox71-kanMX-lox66, *gal4Δ*::HygB | (Zhao et al., 2018) |
| yJDC94 | AY-Y406 CEN/ARS *HIS4*, $P_{REV1}$_WT-TPDNAP1_$T_{ADH1}$ (Ec318) | |
| SAWy118 | yEZ44 *gal80Δ*, *gal4Δ*::HygB | |
| SAWy119 | SAWy118 *his3*::*HIS3*, $P_{TEF1}$_VP16-EL222_$T_{CYC1}$, $P_{C120}$_GAL80_$T_{ADH1}$, $P_{ADH1}$_GAL4_$T_{ACT1}$, $P_{C120}$_GAL80_$T_{ADH1}$ (EZL571) | |
| SAWy145 | SAWy119 *erg9*::$P_{MET3}$-ERG9 | |
| SAWy245 | SAWy119 *leu2*::*LEU2*, $P_{GPD1}$_aaFS_$T_{ADH1}$ (SAWlig232) | |
| SAWy254 | SAWy145 *leu2*::*LEU2*, $P_{GPD1}$_aaFS_$T_{ADH1}$ (SAWlig232) | |
| SAWy264 | SAWy145 *δ*::bleMX6, $P_{GPD1}$_mvaE_$T_{ADH1}$, $P_{TEF}$_mvaS_$T_{ACT1}$, $P_{PGK}$_Acs(L641p)_$T_{CYC1}$ (JMCp10) | |
| SAWy324 | SAWy145 *δ*::bleMX6::$P_{GPD1}$_aaFS_$T_{ADH1}$ (SAWlig260) | |
| SAWy366 | SAWy324 *ura3*::*URA3*, $P_{GPD1}$_mvaE_$T_{ADH1}$, $P_{TEF1}$_mvaS_$T_{ACT1}$, $P_{PGK1}$_Acs(L641p)_$T_{CYC1}$ (SAWlig100) *leu2*::*LEU2*, $P_{GPD1}$_ERG19_$T_{CYC1}$, $P_{TEF1}$_ERG8_$T_{ACT1}$, $P_{PGK1}$_ERG12_$T_{ADH1}$ (SAWlig120) | |
| SAWy518 | SAWy119 *hmg2Δ*::neoMX | |
| SAWy524 OptoMEV | SAWy518 *hmg1*::$P_{C120}$-HMG1 (OptoMEV) | |
| SAWy525 | SAWy524 *leu2*::*LEU2*, $P_{REV1}$_TPDNAP1-4-2_$T_{ADH1}$ (SAWlig424_MUT) | |
| SAWy544 | yJDC94 *p1*::*URA3*, $P_{10B2}$_mNeonGreen_A75_Ribozyme_$T_{ADH1}$ (SAWlig427) | |
| SAWy639 | yJDC94 *p1*::*URA3*, $P_{10B2}$_*JEN1t*_A75_Ribozyme_$T_{ADH1}$ (SAWlig500) | |
| SAWy644 | SAWy524 *jen1Δ*::natMX6 | |
| SAWy678 | Protoplast fusion SAWy525 x SAWy544 (OrthoRep-mNG-mut) | |
| SAWy691 | SAWy524 *leu2*::*LEU2*, $P_{REV1}$_TPDNAP1_$T_{ADH1}$ (SAWlig424_WT) | |
| SAWy696 | Protoplast fusion SAWy691 x SAWy544 (OrthoRep-mNG-wt) | |
| SAWy700 (OptoRep-*JEN1t*) | Protoplast fusion SAWy525 x SAWy639 (OptoRep-*JEN1t*) | |
| SAWy705 | SAWy524 CEN/ARS *URA3*, empty vector (pYZ125) | |

| | | |
|---|---|---|
| SAWy711 | SAWy524 CEN/ARS *URA3*, $P_{GPD1}$_*JEN1t*(WT)_$T_{ADH1}$ (SAWlig523) | |
| SAWy712 Strain $JEN1t^{Y180C}$ | SAWy524 CEN/ARS *URA3*, $P_{GPD1}$_*JEN1t*(Y180C)_$T_{ADH1}$ (SAWlig548) | |
| SAWy715 | SAWy712 ($P_{C120}$-*hmg1Δ*)::natMX6. This deletion removes the OptoMEV genotype/phenotype from the background | |
| SAWy717 | SAWy715 *leu2*::*LEU2*, pTEF1_GFP_aaFS_SSA1t, $P_{GPD1}$_ERG19_ $T_{CYC1}$, $P_{TEF1}$_ERG8_$T_{ACT1}$, $P_{PGK1}$_ERG12_$T_{ADH1}$ (SAWlig568) | |
| SAWy719 | SAWy738 *hmg1Δ*::natMX6. This deletion removes the OptoMEV genotype/phenotype from the background | |
| SAWy720 | SAWy739 *hmg1Δ*::natMX6. This deletion removes the OptoMEV genotype/phenotype from the background | |
| SAWy722 | SAWy719 *leu2*::*LEU2*, $P_{TEF1}$_GFP_aaFS_$T_{SSA1}$, $P_{GPD1}$_ERG19_$T_{CYC1}$, $P_{TEF1}$_ERG8_$T_{ACT1}$, $P_{PGK1}$_ERG12_$T_{ADH1}$ (SAWlig568) | |
| SAWy723 | SAWy720 *leu2*::*LEU2*, $P_{TEF1}$_GFP_aaFS_$T_{SSA1}$, $P_{GPD1}$_ERG19_$T_{CYC1}$, $P_{TEF1}$_ERG8_$T_{ACT1}$, $P_{PGK1}$_ERG12_$T_{ADH1}$ (SAWlig568) | |
| SAWy726 | SAWy324 *leu2*::*LEU2*, $P_{TEF1}$_GFP_aaFS_$T_{SSA1}$, $P_{GPD1}$_ERG19_$T_{CYC1}$, $P_{TEF1}$_ERG8_$T_{ACT1}$, $P_{PGK1}$_ERG12_$T_{ADH1}$ (SAWlig568) | |
| SAWy727 | SAWy324 *leu2*::*LEU2*, $P_{TEF1}$_GFP_aaFS_$T_{SSA1}$, $P_{GPD1}$_ERG19_$T_{CYC1}$, $P_{TEF1}$_ERG8_$T_{ACT1}$, $P_{PGK1}$_ERG12_$T_{ADH1}$, $P_{GPD1}$_JEN1t (Y180G)_$T_{ADH1}$ (SAWlig602) | |
| SAWy728 | SAWy119 *leu2*::*LEU2*, $P_{TEF1}$_GFP_aaFS_$T_{SSA1}$, $P_{GPD1}$_ERG19_$T_{CYC1}$, $P_{TEF1}$_ERG8_$T_{ACT1}$, $P_{PGK1}$_ERG12_$T_{ADH1}$ (SAWlig568) | |
| SAWy729 | SAWy728 CEN/ARS *URA3*, $P_{GPD1}$_JEN1t(Y180C)_$T_{ADH1}$ (SAWlig548) | |
| SAWy730 | SAWy728 CEN/ARS *URA3*, $P_{GPD1}$_JEN1t(Y180G)_$T_{ADH1}$ (SAWlig598) | |
| SAWy731 | SAWy728 CEN/ARS *URA3*, $P_{GPD1}$_JEN1t(Y180A)_$T_{ADH1}$ (SAWlig599) | |
| SAWy733 | SAWy264 *leu2*::*LEU2*, $P_{TEF1}$_GFP_aaFS_$T_{SSA1}$, $P_{GPD1}$_ERG19_$T_{CYC1}$, $P_{TEF1}$_ERG8_$T_{ACT1}$, $P_{PGK1}$_ERG12_$T_{ADH1}$ v(SAWlig568) | |
| SAWy736 | SAWy728 CEN/ARS *URA3*, empty vector (pYZ125) | |
| SAWy738 Strain $JEN1t^{Y180G}$ | SAWy524 CEN/ARS *URA3*, $P_{GPD1}$_JEN1t(Y180G)_$T_{ADH1}$ (SAWlig598) | |

| | | |
|---|---|---|
| SAWy739 Strain *JEN1t*$^{Y180A}$ | SAWy524 CEN/ARS *URA3*, $P_{GPD1}$_JEN1t(Y180A)_$T_{ADH1}$ (SAWlig599) | |
| SAWy740 | SAWy524 + CEN/ARS *URA3*, $P_{GPD1}$_JEN1t(Y180T)_$T_{ADH1}$ (SAWlig600) | |
| SAWy743 | SAWy727 *hmg1Δ*::natMX6 | |
| SAWy749 | SAWy743 trp1:: *TRP1*, $P_{GPD1}$_ERG20_$T_{CYC1}$, $P_{TEF1}$_IDI1_$T_{ACT1}$, $P_{GPD1}$_aaFS_$T_{ADH1}$ | |

**Appendix Table S7.** Relevant primers in this study.

| Primer | Sequence (5' – 3') | Purpose |
|---|---|---|
| SAWpri310 | ATCTGACTTACTTTTACTTAATTGTGTTCTTTCCAAATTAG TTCAACAAGGTTCCCACATACAACCTCAAtacgctgcaggtcgacaacc | HMG2 KO |
| SAWpri311 | CATGCAGATGATTTAGAATAGCTAGACAATACAAAGATA TAAAGTATCACCATGTAAACTACAAGAGccactagtggatctgatatcacc | HMG2KO |
| SAWpri326 | ACTAAGGGCTGGAACATAGTGTATCATTGTCTAATTGTT GATACAAAGTAGATAAATACATAAAACAAGCtacgctgcaggtcgacaacc | HMG1KO |
| SAWpri327 | TACGTAACACATGGTGCTGTTGTGCTTCTTTTTCAAGAG AATACCAATGACGTATGACTAAGTctagtggatctgatatcacctaTACCG | HMG1KO |
| SAWpri606 | AAGAAGAGTAACAGTTTCAAAAGTTTTTCCTCAAAGAG ATTAAATACTGCTACTGAAAATtacgctgcaggtcgacaacc | JEN1KO |
| SAWpri607 | TATATGTGAAATGCAGTTACATAGAGAAGCGAACACGCCCTAGA GAGCAATGAAAAGTGAggccactagtggatctgatatcacc | JEN1KO |
| SAWpri657 | gtctattttacacttttgacctataagtc | p1 |
| SAWpri713 | CTGCTTGCGCTATGGGGTTCATATTTGCTATC | JEN1 Y180C |
| SAWpri714 | ATAGCGCAAGCAGAAAAAAATAGACCTGATAGG | JEN1 Y180C |
| SAWpri761 | TCTGCTNNKGCTATGGGGTTCATATTTGCTATC | JEN1 Y180NNK |
| SAWpri762 | CATAGCKNNAGCAGAAAAAAATAGACCTGATAGG | JEN1 Y180NNK |

## Appendix References


Barata-Antunes, C., Talaia, G., Broutzakis, G., Ribas, D., De Beule, P., Casal, M., Stefan, C. J., Diallinas, G., & Paiva, S. (2022). Interactions of cytosolic tails in the Jen1 carboxylate transporter are critical for trafficking and transport activity. *Journal of Cell Science*, *135*(10). https://doi.org/10.1242/JCS.260059/275079/

Entian, K. D., & Kötter, P. (2007). 25 Yeast Genetic Strain and Plasmid Collections. *Methods in Microbiology*, *36*, 629–666. https://doi.org/10.1016/S0580-9517(06)36025-4

Kichuk, T., Dhamankar, S., Malani, S., Hofstadter, W. A., Wegner, S. A., Cristea, I. M., & Avalos Correspondence, J. L. (2024). Article Using MitER for 3D analysis of mitochondrial morphology and ER contacts. *Cell Reports Methods*, *4*, 100692. https://doi.org/10.1016/j.crmeth.2023.100692

Ravikumar, A., Arzumanyan, G. A., Obadi, M. K. A., Javanpour, A. A., & Liu, C. C. (2018). Scalable, Continuous Evolution of Genes at Mutation Rates above Genomic Error Thresholds. *Cell*, *175*(7), 1946-1957.e13. https://doi.org/10.1016/J.CELL.2018.10.021

Wegner, S. A., Chen, J. M., Ip, S. S., Zhang, Y., Dugar, D., & Avalos, J. L. (2021). Engineering acetyl-CoA supply and ERG9 repression to enhance mevalonate production in Saccharomyces cerevisiae. *Journal of Industrial Microbiology & Biotechnology*, *48*(9–10). https://doi.org/10.1093/JIMB/KUAB050

Zhao, E. M., Lalwani, M. A., Lovelett, R. J., Garciá-Echauri, S. A., Hoffman, S. M., Gonzalez, C. L., Toettcher, J. E., Kevrekidis, I. G., & Avalos, J. L. (2020). Design and Characterization of Rapid Optogenetic Circuits for Dynamic Control in Yeast Metabolic Engineering. *ACS Synthetic Biology*, *9*(12), 3254–3266. https://doi.org/10.1021/ACSSYNBIO.0C00305

Zhao, E. M., Zhang, Y., Mehl, J., Park, H., Lalwani, M. A., Toettcher, J. E., & Avalos, J. L. (2018). Optogenetic regulation of engineered cellular metabolism for microbial chemical production. *Nature 2018 555:7698*, *555*(7698), 683–687. https://doi.org/10.1038/nature26141
