# Supplementary material for: Orthogonal replication with optogenetic selection evolves yeast JEN1 into a mevalonate transporter
Source: Mol Syst Biol. 2025 Jun 11;21(9):1190–213. doi: 10.1038/s44320-025-00113-5 (PMC12405511; doi:10.1038/s44320-025-00113-5)
Supplement: Supplementary file 8 — Expanded View Figures [file 44320_2025_113_MOESM8_ESM.pdf]

Expanded View Figures

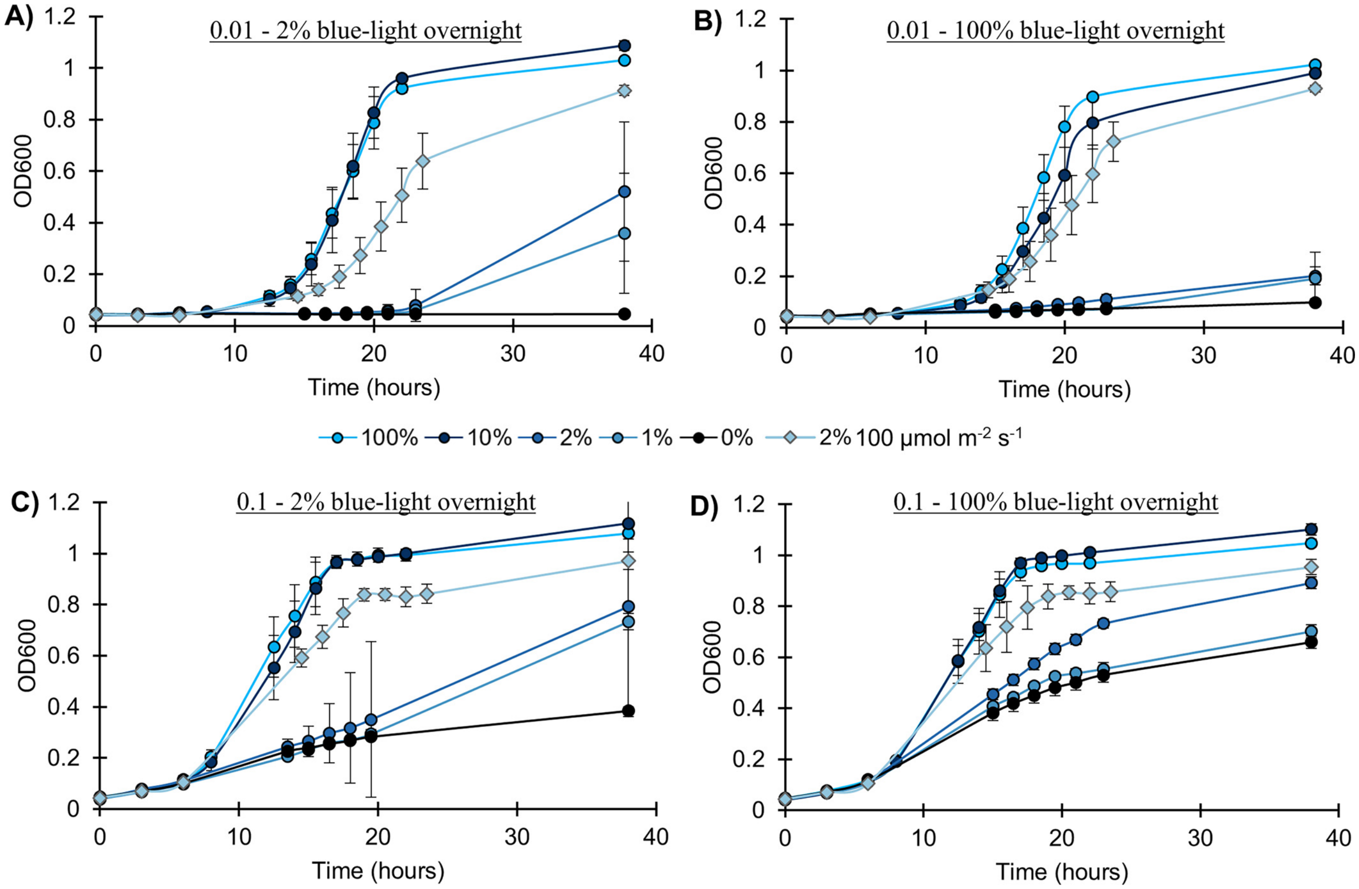

Figure EV1. OptoMEV growth in different light doses and starting cell densities.

(**A**, **B**) Cultures of the OptoRep-JEN1t strain (SAWy700) inoculated at an initial $OD_{600} = 0.01$ from overnight cultures grown in either (**A**) 2% light (2 s on/100 s total) or (**B**) 100% light, and then incubated to grow under different light conditions. (**C**, **D**) Cultures of the OptoRep-JEN1t strain (SAWy700) inoculated at an initial $OD_{600} = 0.1$ from overnight cultures grown in either (**C**) 2% light (2 s on/100 s total) or (**D**) 100% light, and then incubated to grow under different light conditions. Optical density measurements are reported in Tecan absorbance units (see "Methods"). Where not explicitly shown, light intensity varied between 40 and 60 μmol m^{-2} s^{-1}. For all conditions light intensity in the overnight was equivalent to intensities in the experiment (i.e., between 40 and 60 μmol m^{-2} s^{-1} except for the 100 μmol m^{-2} s^{-1} condition). Average and individual data points for 4 biologically independent replicates are shown with standard deviation for each measured time point.

 © The Author(s)

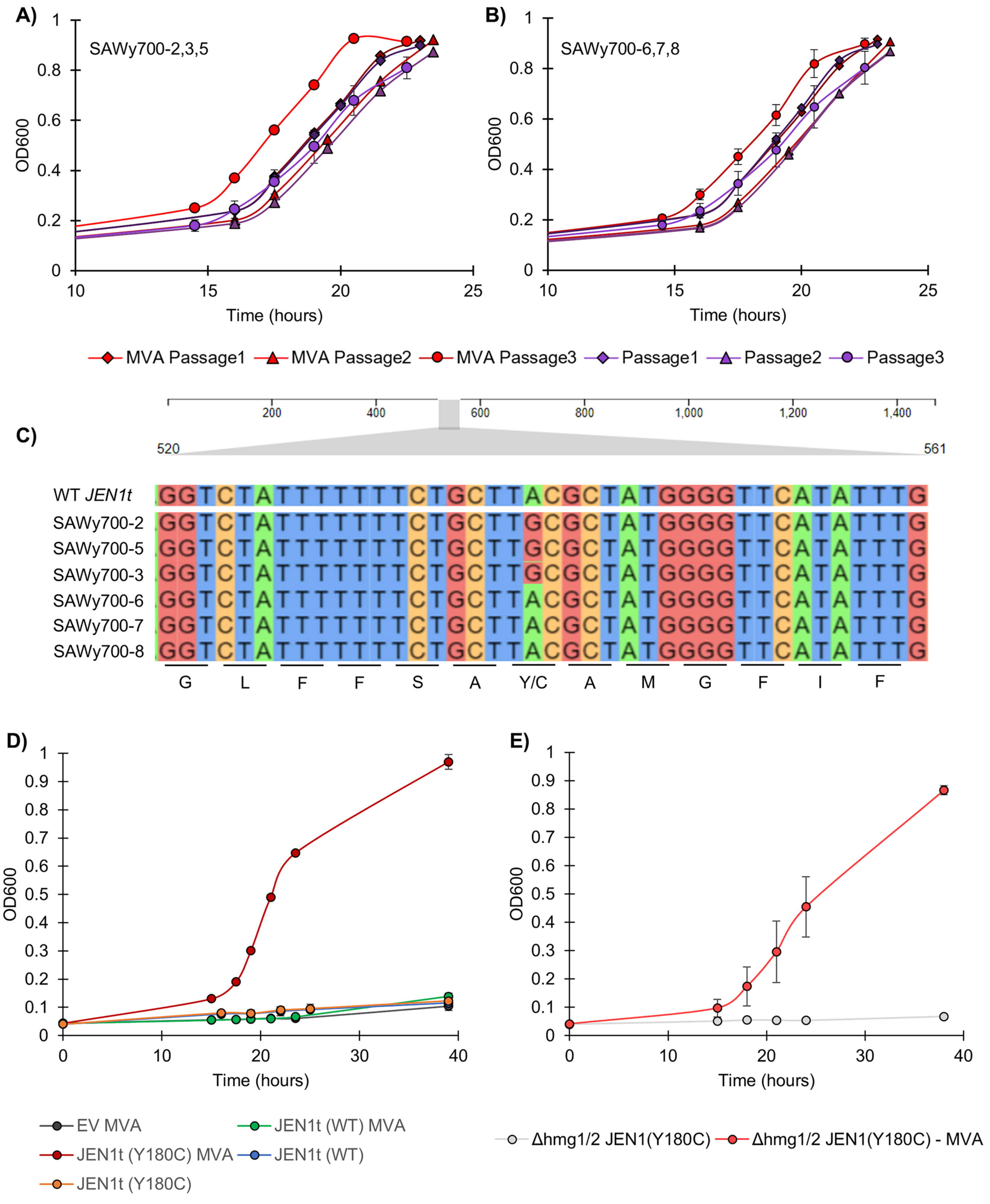
A)
SAWy700-2,3,5
OD600
Time (hours)
B)
SAWy700-6,7,8
OD600
Time (hours)
MVA Passage1
MVA Passage2
MVA Passage3
Passage1
Passage2
Passage3
C)
520
561
WT JEN1t GGTCTATTTTTTTCTGCTTACGCTATGGGGTTCATATTTG
SAWy700-2 GGTCTATTTTTTTCTGCTTGCGCTATGGGGTTCATATTTG
SAWy700-5 GGTCTATTTTTTTCTGCTTGCGCTATGGGGTTCATATTTG
SAWy700-3 GGTCTATTTTTTTCTGCTTGCGCTATGGGGTTCATATTTG
SAWy700-6 GGTCTATTTTTTTCTGCTTACGCTATGGGGTTCATATTTG
SAWy700-7 GGTCTATTTTTTTCTGCTTACGCTATGGGGTTCATATTTG
SAWy700-8 GGTCTATTTTTTTCTGCTTACGCTATGGGGTTCATATTTG
G L F F S A Y/C A M G F I F
D)
OD600
Time (hours)
EV MVA
JEN1t (WT) MVA
JEN1t (Y180C) MVA
JEN1t (WT)
JEN1t (Y180C)
E)
OD600
Time (hours)
Δhmg1/2 JEN1(Y180C)
Δhmg1/2 JEN1(Y180C) - MVA

© The Author(s)

Figure EV2. OptoRep-*JEN1t* lineages with improved growth in mevalonate and *JEN1t* mutations obtained with OptoRep.

(**A**) Averaged growth profiles of SAWy700-2, SAWy700-3, and SAWy700-5, from which *JEN1t*^{Y180C} evolved independently three times, show a significant decrease in lag relative to (**B**) the averaged growth profiles of SAWy700-6, SAWy700-7, and SAWy700-8, from which no mutant sequence was identified. MVA refers to growth conditions supplemented with 10 mM mevalonate. Data points for (**A**, **B**) show the mean for the measured OD_{600} of the three described lineages, with the standard deviation only the third passage shown (some error bars are smaller than the data point icons). (**C**) Individual colonies were isolated after purifying selection and their p1 plasmids sequenced, resulting in only one and the same transition mutation A539G (corresponding to A818 of wild-type full-length *JEN1*), equating in a Y180C mutation in the *JEN1t* amino acid sequence (corresponding to Y273 in the full-length *JEN1* protein). Mutations which occurred in at least one of the sequenced colonies are reported. (**D**) The *JEN1t*^{Y180C} mutation was expressed in the OptoMEV strain using a CEN/ARS plasmid and assayed for mevalonate-dependent growth (MVA—10 mM mevalonate) in nonpermissive conditions (complete darkness). The growth curve was compared to a strain containing the wild-type *JEN1t* ((WT) MVA; SAWy711), an empty vector (EV MVA; SAWy705), or the strain expressing *JEN1t*^{Y180C} (SAWy712) but incubated in medium without mevalonate. Conditions without MVA refer to unsupplemented medium. (**E**) The mevalonate-dependent growth of an HMG-CoA reductase-null genetic background (Δ*hmg1*/Δ*hmg2*), expressing the *JEN1t*^{Y180C} transporter (SAWy715). Optical density measurements are reported in Tecan absorbance units (see "Methods"). The average for 4 biologically independent replicates are shown with standard deviation (some error bars are smaller than the data point icons) for each measured time point in (**D**, **E**).

 © The Author(s)

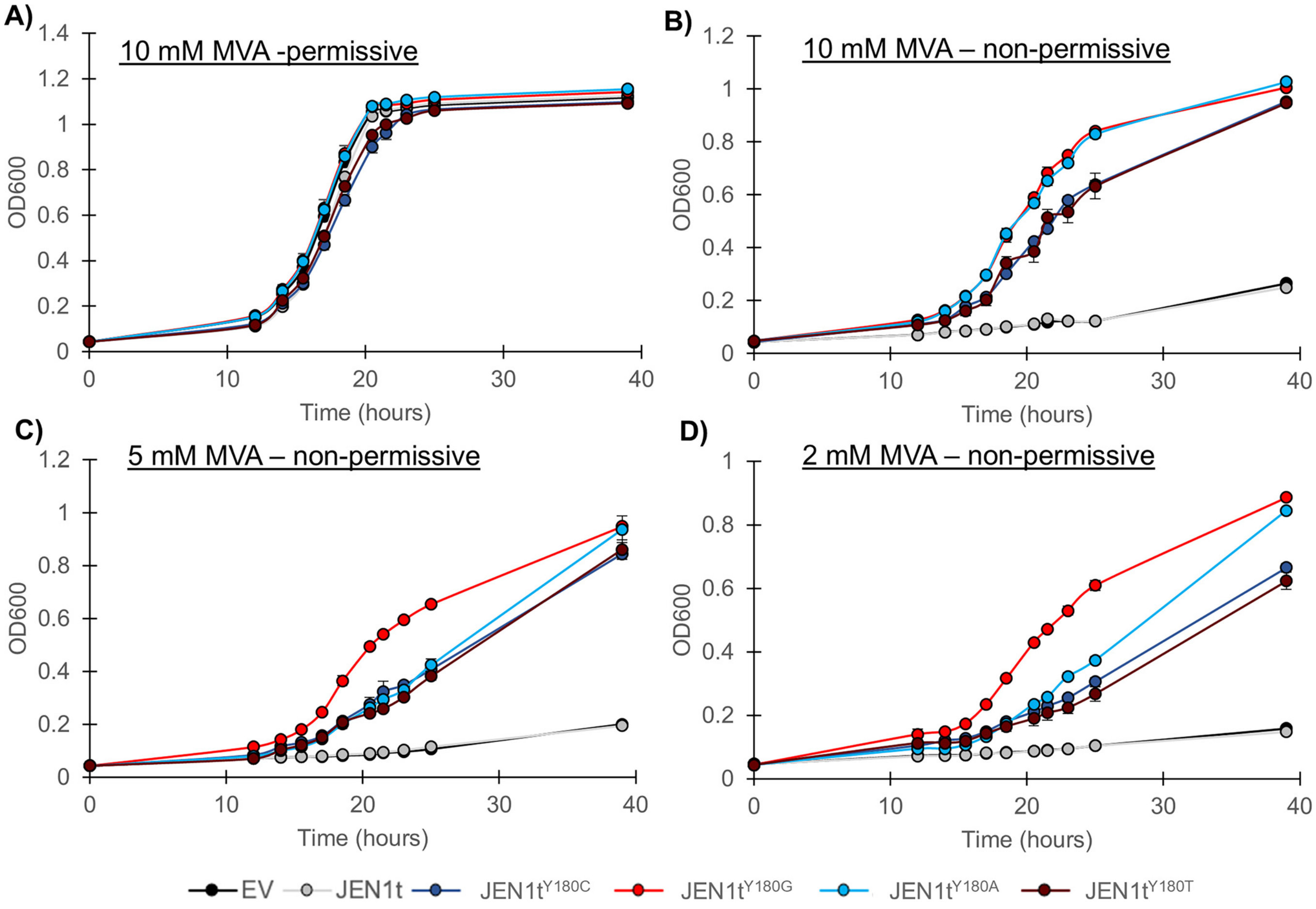

Figure EV3. Validation of Y180 NNK library hits.

(**A–D**) The observed mutations plus controls were introduced into the OptoMEV strain (Empty Vector: SAWy705, *JEN1t*^{Y180}: SAWy711, *JEN1t*^{Y180C}: SAWy712, *JEN1t*^{Y180G}: SAWy738; *JEN1t*^{Y180A}: SAWy739, *JEN1t*^{Y180T}: SAWy740) and growth was assayed under different mevalonate (MVA) concentrations as listed. Permissive blue light intensity was ~40 µmol m^{-2} s^{-1}. Optical density measurements are reported in Tecan absorbance units (see "Methods"). Average and individual data points for 4 biologically independent replicates are shown with standard deviation for each measured time point.

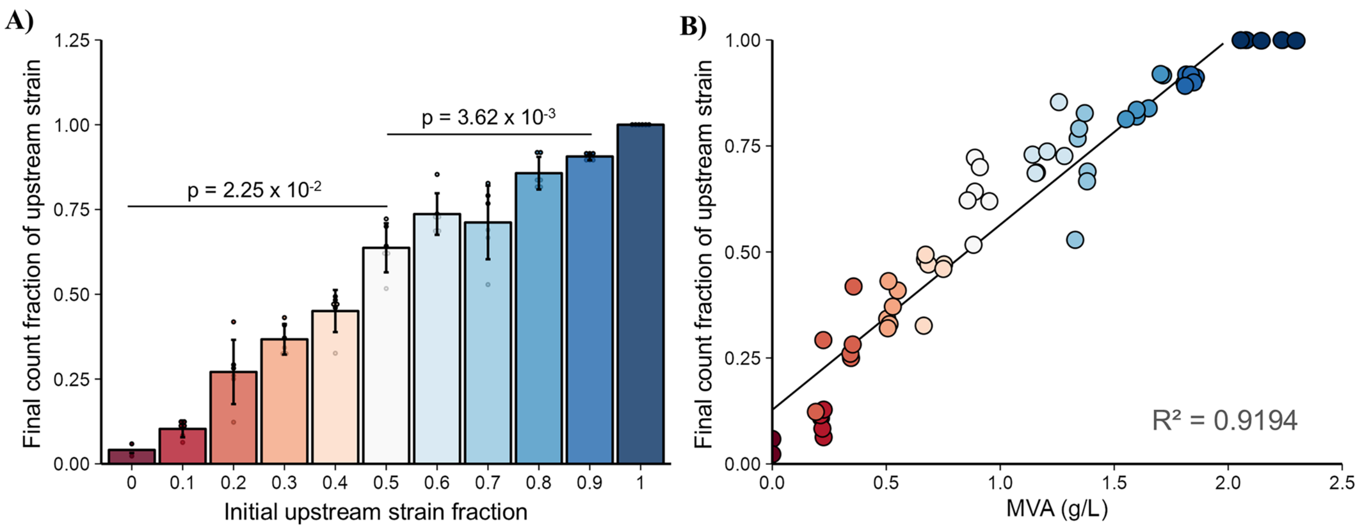

Figure EV4. Final compositions of mevalonate-exchange consortia for farnesene production.

(**A**) The final count fraction of the upstream strain (SAWy733) from consortia initiated with different initial fractions of the upstream strain. Serial dilutions for colony counting were performed post-fermentation with selection for either the downstream strain (SAWy749; SC-HIS-LEU-MET-TRP) or both consortia members combined (SC-HIS-LEU-MET). (**B**) correlation between the final colony count fraction of upstream strain and mevalonate (MVA) production by the consortia, with the Pearson coefficient of determination shown. Points are colored according to the initial upstream strain fraction of the consortia (same as **A**). Average and individual data points for 6 biologically independent replicates are shown with error bars showing the standard deviation. Data analysis was performed by Kruskal–Wallis with Dunn's post hoc comparisons for all panels.

 © The Author(s)
